# Supplementary material for: DegQ is an important policing link between quorum sensing and regulated adaptative traits in Bacillus subtilis
Source: Microbiol Spectr. 2023 Sep 7;11(5):e00908-23. doi: 10.1128/spectrum.00908-23 (PMC10581247; doi:10.1128/spectrum.00908-23)
Supplement: Figures S1 to S5 [file spectrum.00908-23-s0001.docx]

**Supplementary Information**

**Figure legends**

**Figure S1:** Allelic variation of P*_degQ_* from different *B. subtilis* wild-type and mutant strains. The partial sequences were aligned using MUSCLE.

**Figure S2:** Expression derived from P*_srfAA_-yfp* construct (fluorescence intensity normalized per optical density at 650 nm) of *Bacillus subtilis* PS-216 wild type strain and its mutants without (white columns) and with (grey columns) the *amyE*::P*_hyperspank_*-*degQ* construct incubated in CM at 37 °C and 200 rpm for 6 h. CM media with mutant strain *comQ::kan* were supplemented with ComX - conditioned 5 % (V/V) M9 minimal medium, where ComX is produced heterologously in *E. coli* ED367 after IPTG induction. Strains carrying *amyE*::P*_hyperspank_*-*degQ* construct were also supplemented with 0.1 mM IPTG. The values presented are means and standard errors (n=3). Different letters above the columns indicate a statistically significant difference (p < 0.05) between mean values across all strains and treatments (i.e. across all columns).

**Figure S3:** Transcriptional levels of *comP* and *comA* genes in *B. subtilis* PS-216 Δ*comQ* (white columns) and *comQ::kan* (gray columns) mutant strains compared to *B. subtilis* PS-216 wild-type strain. Cells were grown in CM medium at 37 °C and 200 rpm until stationary phase. Values are presented as means and standard errors (*n*=4). Different letters above the columns indicate a statistically significant difference (p < 0.05) between mean values across all strains and treatments (i.e. across all columns).

**Figure S4:** Expression derived from the P*_srfAA_-yfp* construct (fluorescence intensity normalized per optical density at 650 nm) of *B. subtilis* PS-216 wild type strain and its mutants without (white columns) and with (grey columns) the *amyE*::P*_hyperspank_*-*comP* construct following growth in
CM medium at 37 °C and 200 rpm for 6 h. CM media with mutant strains Δ*comQ* and *comQ::kan* were supplemented with ComX – conditioned 5 % (V/V) M9 minimal medium, where ComX is produced heterologously in *E. coli* ED367 after IPTG induction. Strains carrying the *amyE*::P*_hyperspank_*-*comP* construct were also supplemented with 0.1 mM IPTG. The values presented are means and standard errors (n=3). Different letters above the columns indicate a statistically significant difference (p < 0.05) between mean values across all strains and treatments (i.e. across all columns).

**Figure S5:** Growth of *Bacillus subtilis* PS-216 wild type strain and its mutants during static incubation in MSgg medium at 37 °C without ComX (A and C) and with ComX - conditioned 20 % (V/V) M9 minimal medium, where ComX is heterologously produced in *E. coli* ED367 after IPTG induction (B and D). Exogenous ComX was added to the cultures 8 hours after static incubation in MSgg medium. The values presented are means and standard errors (n=3).

**Figure S1**

**Figure S2**

**Figure S3**

**Figure S4**

**Figure S5**
